# Supplementary material for: FluxPyt: a Python-based free and open-source software for 13C-metabolic flux analyses
Source: PeerJ. 2018 Apr 27;6:e4716. doi: 10.7717/peerj.4716 (PMC5933345; doi:10.7717/peerj.4716)
Supplement: Supplemental Information 2 [file peerj-06-4716-s002.pdf]

FluxPyt User Guide

Installation (for Windows):

Download and install Anaconda distribution (version 3.4.4.0 was used in this tutorial).

Open an Anaconda command prompt.

Click on Windows start button.

Type 'anaconda'. A link to open Anaconda command prompt will be shown.

Type following commands to create new environment in Anaconda for running FluxPyt:

```
conda create -n FluxPyt_env python=3.6.1 numpy=1.12.1 scipy=0.19.0 sympy=1.0

activate FluxPyt_env

conda install -c conda-forge lmfit

conda install -c sjpfenninger glpk

conda install csvkit=0.9.1

conda install matplotlib=2.0.2

conda install pandas=0.20.1

conda install spyder

pip install fluxpyt
```

Installation (for Linux):

Download and install Anaconda distribution. (See https://docs.anaconda.com/anaconda/install/linux for installation instructions.)

Open a Terminal.

Type following commands to create new environment in Anaconda for running FluxPyt:

```
conda create -n FluxPyt_env python=3.6.1 numpy=1.12.1 scipy=0.19.0 sympy=1.0
```

```
source activate FluxPyt_env

conda install -c conda-forge lmfit

conda install -c bioconda glpk

conda install csvkit=0.9.1

conda install matplotlib=2.0.2

conda install pandas=0.20.1

conda install spyder

pip install fluxpyt
```

Model construction:

1. Open Microsoft Excel or LibreOfffice Calc.

2. Starting from first column, write the labels of each column of the model as follows:

RxnID	rxnEq	atom_transition	rates	rxnType	basis	deviation

3. Write reaction, atom transition equations, rates, reaction types, basis fluxes and deviations as follows:

RxnID	rxnEq	atom_transition	rates	rxnType	basis	deviation
V01	PYR_EX -> PYR	abc -> abc		F	1	
V17	AKG -> AKG_B			B	0.23	0.01
V19	PYR + PYR -> VALX + CO2X	abc + def -> abefc + d	1	S		

4. Type of reactions:

Reaction type	Description
F	Irreversible reaction used for metabolite and isotopomer balance.
FR	Forward reaction of reversible reaction used for metabolite and isotopomer balance.
R	Reversible reaction used for metabolite and isotopomer balance
S	Reaction used for isotopomer balancing. Rate of this reaction should be kept equal to 1.
B	Irreversible reaction which is not used for only metabolite balance.

5. Put '*' sign in 8th column in row corresponding to the reaction which the user wants to take as basis. The basis flux is usually the major substrate uptake flux, e.g. glucose influx reaction.
6. Atom transition equation:
   a. The order of metabolites in reaction equation and respective atom composition in atom transition equation should be same.
   b. Metabolites e.g. ATP, that do not take part in isotopomer balance equations should be represented by 'X' in atom transition equation. Following is the representation:

V10	PYR + CO2 + ATP -> OAA	abc + d + X -> abcd		F			

7. After writing all the reactions leave one row empty and write excluded metabolites list as follows:

##	excludedMetabolites
#	PYR_EX

#	GLU_EX

- The "##" for exclude metabolite list and all subsequent identifiers should be in first column of the spreadsheet.
- The excluded metabolites are the end metabolites in the metabolic network which are not included in the stoichiometric matrix.

8. Add input substrate and simulated MDVs (Mass Distribution Vectors) under the identifiers, 'inputSubstrates' and 'measuredIsotopes' respectively.

9. Leave one row empty and write measurement and error values as follows:

##	measurements	errors
#	0.02983	0.0003
#	0.24378	0.00244
#	0.05892	0.00059

10. Save file as comma delimited file (.csv). You can give any name to the file but the extension should be ".csv", e.g. "myModel.csv".

Substrate input File

1. Beginning from first row and column, substrate information should be written as follows:

Substrate	labeling pattern	ratio
PYR_EX	0.99 0.0107 0.0107	0.5
PYR_EX	0.99 0.99 0.99	0.5
GLU_EX	0.99 0.0107 0.0107 0.0107 0.0107	1

2. The first column should be the substrate ID. This should be same as the metabolite ID of the substrate as written in reaction equations.

3. Second column represents carbon label at respective carbon atoms of the substrate molecule. In above example, value of 0.99 represents 99% of the carbons at that position is labelled. This value may vary based on the purity of labelled substrate you use in your experiment. The value of 0.0107 represents the natural abundance of ^{13}C carbon.

4. Third column represents ratio of specific labelled substrate used. If mixture of same metabolite labelled at different carbons is used, there proportion has to be written. In above case, 1-^{13}C-pyruvate and U-^{13}C-pyruvate is used in equal proportions. The proportion of Glutamate is written as 1 as there is only one form of glutamate used, i.e. 1-^{13}C-glutamate. Similarly, if the proportion of different forms of a metabolite used is unknown, then value of "1" should be written under the "ratio" column and separate uptake reactions for each form of the metabolite should be written in the model.
5. Save file as comma delimited file named as "substrate_input.csv"

MID correction File:

1. Data for correction of simulated MIDs is included to calculate simulated MIDS considering label incorporation due to natural abundance of ^{13}C-carbon. In cases where natural isotope correction is not necessary, this file is used to extract truncated MID vectors from simulated MIDs if truncated MID vector is used as the measurement data.
2. Following is an example how correction file is written:

ALAX:111	C8H26N1O2Si2	3	4
VALX:11111	C8H30N1O2Si2	4	6

3. There is no header row in this file.
4. First column is the measured mass isotopomer. Read Antoniewicz et al. 2007 [1] for details about amino acids, their molecular formulae.
5. Second column is the molecular formula of the amino acid derivative excluding the carbon backbone.
6. Third column represents number of masses include in the model for the amino acid.
7. Forth column represents the EMU size plus 1 of the mass isotopomer.
8. Save the file as 'corr_file.csv'.

How to use FluxPyt?

User guide is written for Anaconda installation in windows.

1. Make a folder (e.g. "mymodel") wherein you will store the csv files of model, substrate and correction data. All the three files should be present in same folder.
2. Open spyder window.
    a. Open anaconda console window by typing "Anaconda" in search program panel in Windows.
    b. Activate "FluxPyt_env" environment by typing:

        activate FluxPyt_env

    c. In the console window, type "spyder" and press "Enter"

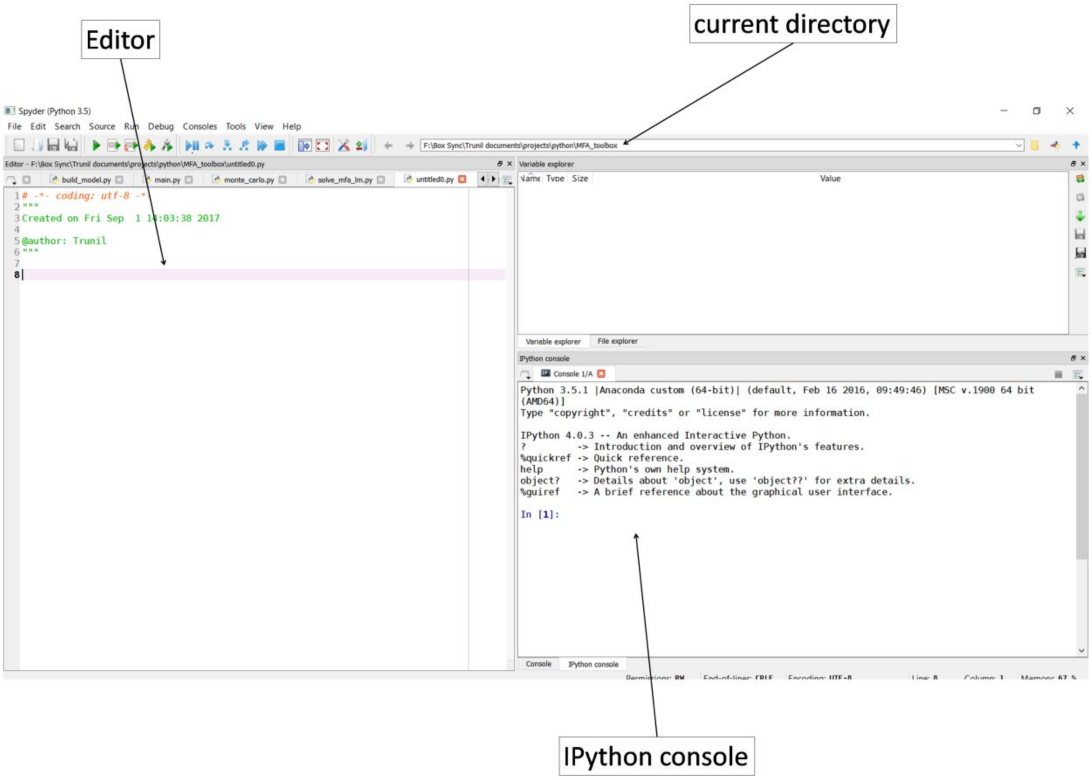

Figure: Screenshot of spyder interactive development environment (IDE)

3. In the “current directory” pane, paste the path to the “mymodel” folder and press “Enter”. For example, if you have stored the “mymodel” folder in “E” drive of the computer inside a folder named as “MFA_analysis”, then the path will be:

   “E:\MFA_analysis\mymodel”

   This can also be done by following way:

   ```
   import  os
   os.chdir('E:\\MFA_analysis\\mymodel')
   ```

4. Flux estimation:
   a. In IPython console, type following.

      ```
      import  fluxpyt

      fluxpyt.main()
      ```

   b. Python will ask, “Enter file name:”. Type the name of the model you wrote and press “Enter”. Do not write the extension of the file i.e. “.csv”. It will be automatically added to filepath once the program runs.

      ```
      myFile
      ```

   c. Python will ask, “Is data for natural isotope correction available (y/n)?”. Type ‘y’ if correction for natural isotopes is required. Press “Enter”.
   d. Python will ask, “Enter Number of Iterations”. It is recommended to use at least 10 iterations.
   e. The flux estimation will run. If there are no errors or exceptions, optimal fluxes and rough estimates of flux ranges will be calculated.
   f. After completion of flux estimation FluxPyt will ask:

      ```
      Optimization completed.

      Is network diagram template available? (y/n)
      ```

g. Type ‘y’ if yes. Note that the flux diagram template diagram filename should be same as the model filename.
h. FluxPyt will again ask if Monte_Carlo simulations are to be run or not. If yes, it will ask number of data sets to be generated. Default value is 500. If no, then the program will exit.
i. One can Monte Carlo simulations and bootstrapping (provided Monte Carlo simulation has previously completed) separately.

5. Monte Carlo and bootstrapping
    a. Open spyder and make the current directory to model folder where flux estimation had been carried out.
    b. Type following.

```
from fluxpyt import monte_carlo

monte_carlo()
```

    c. Boot strapping will be automatically done. If you want to perform bootstrapping independently then use following commands.

```
from fluxpyt  import bootstrap

bootstrap()
```

In case the user wants to perform bootstrap analysis for specific reactions, for example, V02 and V06, use following commands.

```
from fluxpyt  import bootstrap

list_of_rxns = [‘V02’, ‘V06’]

bootstrap(rxnList = list_of_rxns)
```

Output Files

FluxPyt generates several result files for use during MFA (Table 1). FluxPyt provides automatic generation of flux maps based on template, graph representing differences in measured and calculated MIDs and the box plots representing bootstrap analysis results. All these results are also written in tabular form as separate csv files for further analysis by the user. In addition, all results are stored as pickle files in case the user wants to analyze the results in Python. After the Monte Carlo analysis has been done, user can do bootstrap analysis for reactions that he chooses (See point No. 5c in “How to use FluxPyt?” section).

Table 1: Output files generated by FluxPyt.

File name	Description
**optimal_solution.csv**	Lists the optimal flux values for each reaction calculated by FluxPyt. The sum of squared residue and the chi-square cut-off valuesare also written here.
**optimization_data.pckl**	Consists of the optimization result, and the data required for calculation of the confidence intervals by Monte Carlo method.
**calculated_mids.pckl**	Contains calculated MIDs from the last iteration. It is used to write the "calulated_mids.txt" at the end of optimization.
**calulated_mids.txt**	Contains the calculated MIDs at the optimal solution.
**mid_dev.png**	A graph showing the differences in the calculated and the measured MIDs at the optimal solution.
**modelName_fluxMap.svg**	The fluxmap drawn from a template (e.g.,modelName.svg) with optimal flux values.
**montecarlo_results.csv**	Contains the results of Monte Carlo analysis. For each reaction, the optimal solution and the 2.5, 16, 50, 84 and 97.5 percentile values are written.
**flux_std.pckl**	Contains the results of Monte Carlo analysis which is used by the bootstrap function.
**bootstrap_dataframe.pckl**	Contains bootstrap results as apandasdataframe. Each column contains the raw data from the bootstrap analysis for a particular reaction.
**confidence_intervals_bootstrap.csv**	A list of 68% and 95% confidence intervals of the flux through each reaction calculated by the bootstrap analysis.
**monte_carlo_fig.png**	Box plots representing confidence intervals calculated by bootstrap analysis.
