# Supplementary material for: FluxPyt: a Python-based free and open-source software for 13C-metabolic flux analyses
Source: PeerJ. 2018 Apr 27;6:e4716. doi: 10.7717/peerj.4716 (PMC5933345; doi:10.7717/peerj.4716)
Supplement: Supplemental Information 3 [file peerj-06-4716-s003.pdf]

```
Python 3.6.1 |Anaconda custom (64-bit)| (default, May 11 2017, 13:25:24) [MSC v.
1900 64 bit (AMD64)]
Type "copyright", "credits" or "license" for more information.

IPython 5.3.0 -- An enhanced Interactive Python.
?         -> Introduction and overview of IPython's features.
%quickref -> Quick reference.
help      -> Python's own help system.
object?   -> Details about 'object', use 'object??' for extra details.

In [1]:

In [1]: import fluxpyt

In [2]: fluxpyt.main()
Enter file name:

tca



Is data for natural isotope correction available (y/n)?

n



Enter Number of Iterations

10
```
