# Supplementary material for: FluxPyt: a Python-based free and open-source software for 13C-metabolic flux analyses
Source: PeerJ. 2018 Apr 27;6:e4716. doi: 10.7717/peerj.4716 (PMC5933345; doi:10.7717/peerj.4716)
Supplement: Supplemental Information 4 [file peerj-06-4716-s004.docx]

| File name | Description |
| --- | --- |
| optimal_solution.csv | Lists the optimal flux values for each reaction calculated by FluxPyt. The sum of squared residue and the chi-square cut-off values are also written here. |
| optimization_data.pckl | Consists of the optimization result, and the data required for calculation of the confidence intervals by Monte Carlo method. |
| calculated_mids.pckl | Contains calculated MIDs from the last iteration. It is used to write the "calulated_mids.txt" at the end of optimization. |
| calulated_mids.txt | Contains the calculated MIDs at the optimal solution. |
| mid_dev.png | A graph showing the differences in the calculated and the measured MIDs at the optimal solution. |
| modelName_fluxMap.svg | The flux map drawn from a template (e.g., modelName.svg) with optimal flux values. |
| montecarlo_results.csv | Contains the results of Monte Carlo analysis. For each reaction, the optimal solution and the 2.5, 16, 50, 84 and 97.5 percentile values are written. |
| flux_std.pckl | Contains the results of Monte Carlo analysis which is used by the bootstrap function. |
| bootstrap_dataframe.pckl | Contains bootstrap results as a pandas dataframe. Each column contains the raw data from the bootstrap analysis for a particular reaction. |
| confidence_intervals_bootstrap.csv | A list of 68% and 95% confidence intervals of the flux through each reaction calculated by the bootstrap analysis. |
| monte_carlo_fig.png | Box plots representing confidence intervals calculated by bootstrap  analysis. |
