# Supplementary material for: FluxPyt: a Python-based free and open-source software for 13C-metabolic flux analyses
Source: PeerJ. 2018 Apr 27;6:e4716. doi: 10.7717/peerj.4716 (PMC5933345; doi:10.7717/peerj.4716)
Supplement: Supplemental Information 6 [file peerj-06-4716-s006.docx]

|  |  | FluxPyt optimal solution |
| --- | --- | --- |
| R01 | GLC_EX -> GLC6P | 106.00 |
| R02 | GLC6P -> TREM | 1.52 |
| R03 | GLC6P -> F6P | 118.24 |
| R04 | F6P -> GLC6P | 63.53 |
| R05 | F6P -> F16BP | 79.51 |
| R06 | F16BP -> DHAP + G3P | 79.51 |
| R07 | DHAP -> G3P | 79.51 |
| R08 | GLC6P -> P5P + CO2 | 48.25 |
| R09 | P5P + P5P -> S7P + G3P | 34.85 |
| R10 | S7P + G3P -> P5P + P5P | 20.20 |
| R11 | S7P + G3P -> E4P + F6P | 44.28 |
| R12 | E4P + F6P -> S7P + G3P | 29.64 |
| R13 | E4P + P5P -> F6P + G3P | 36.49 |
| R14 | F6P + G3P -> E4P + P5P | 24.05 |
| R15 | G3P -> 3PG | 170.87 |
| R16 | 3PG -> PYR | 159.91 |
| R17 | PYR -> ACCOA + CO2 | 89.65 |
| R18 | ACCOA + OAA -> CIT | 65.46 |
| R19 | CIT -> AKG + CO2 | 65.46 |
| R20 | AKG -> 0.5 SUC + 0.5 SUC + CO2 | 54.58 |
| R21 | SUC -> OAA | 54.58 |
| R22 | PYR + CO2 -> OAA | 64.10 |
| R23 | OAA -> PYR + CO2 | 29.57 |
| R24 | OAA + PYR -> LYS + CO2 | 10.89 |
| R25 | 3PG -> SER | 0.19 |
| R26 | SER -> GLY + MTHF | 0.19 |
| R27 | PYR -> LAC | 0.09 |
| R28 | OAA -> THR | 0.02 |
| R29 | THR -> GLY + ACETAL | 0.02 |
| R30 | CO2 -> CO2_EX | 315.26 |
| R31 | CO2_EX -> CO2 | 67.38 |
| R32 | LYS -> LYS_EX | 8.19 |
| R33 | LYS -> LYSDAP_B | 2.71 |
| R34 | GLY -> GLY_EX | 0.21 |
| R35 | TREM + TREM -> TRE_EX | 0.76 |
| R36 | AKG -> AKG_EX | 1.72 |
| R37 | AKG -> AKG_B | 9.16 |
| R38 | ACETAL -> ACETAL_EX | 0.02 |
| R39 | LAC -> LAC_EX | 0.09 |
| R40 | GLC6P -> GLC6P_B | 1.52 |
| R41 | F6P -> F6P_B | 2.27 |
| R42 | P5P -> P5P_B | 6.53 |
| R43 | E4P -> E4P_B | 2.21 |
| R44 | G3P -> G3P_B | 1.04 |
| R45 | 3PG -> 3PG_B | 10.76 |
| R46 | PYR -> PYR_B | 25.21 |
| R47 | OAA -> OAA_B | 12.74 |
| R48 | ACCOA -> ACCOA_B | 24.19 |
| R49 | PYR_B + PYR_B -> VALX + CO2 | 2.36 |
| R50 | E4P_B + PYR_B -> SHKM | 2.21 |
| R51 | SHKM + PYR_B -> CHRM | 2.21 |
| R52 | CHRM -> PHEX + CO2 | 1.08 |
| R53 | CHRM -> TYRX + CO2 | 0.67 |
| R54 | CHRM -> ANTHR + PYR | 0.45 |
| R55 | ANTHR + P5P_B -> CPADR5P | 0.45 |
| R56 | CPADR5P -> INDG + CO2 | 0.45 |
| R57 | INDG -> IND + G3P | 0.45 |
| R58 | IND + 3PG_B -> TRPX | 0.45 |
| R59 | PYR_B + OAA_B -> ILEX + CO2 | 1.68 |
| R60 | PYR_B + PYR_B -> ISV + CO2 | 3.66 |
| R61 | ISV + ACCOA_B -> LEUX + CO2 | 3.66 |
| R62 | P5P_B -> P5P_BT | 6.08 |
| R64 | PYR_B -> PYR_BT | 7.07 |
| R65 | OAA_B -> OAA_BT | 11.06 |
| R66 | ACCOA_B -> ACCOA_BT | 20.53 |
| R67 | 3PG_B -> 3PG_BT | 10.31 |
| R68 | 0.202 ILEX + 0.44 LEUX + 0.13 PHEX + 0.054 TRPX + 0.081 TYRX + 0.284 VALX -> BIOMASS | 8.32 |
