# Supplementary material for: FluxPyt: a Python-based free and open-source software for 13C-metabolic flux analyses
Source: PeerJ. 2018 Apr 27;6:e4716. doi: 10.7717/peerj.4716 (PMC5933345; doi:10.7717/peerj.4716)
Supplement: Supplemental Information 7 [file peerj-06-4716-s007.docx]

| rxnID | -95% CI | -68% CI | median | +68% CI | +95% CI |
| --- | --- | --- | --- | --- | --- |
| R01 | 94.00 | 97.80 | 105.65 | 106.00 | 106.00 |
| R02 | 1.52 | 1.52 | 1.52 | 2.00 | 2.00 |
| R03 | 100.23 | 108.50 | 116.39 | 124.36 | 132.19 |
| R04 | 48.42 | 55.39 | 63.03 | 71.20 | 79.52 |
| R05 | 68.52 | 72.26 | 78.34 | 79.75 | 80.68 |
| R06 | 68.52 | 72.26 | 78.35 | 79.75 | 80.68 |
| R07 | 68.50 | 72.02 | 78.27 | 79.75 | 80.68 |
| R08 | 39.26 | 42.70 | 47.03 | 49.94 | 51.73 |
| R09 | 12.46 | 21.90 | 41.96 | 61.25 | 1505.27 |
| R10 | 0.00 | 8.09 | 27.60 | 46.28 | 1492.42 |
| R11 | 15.96 | 27.49 | 39.62 | 58.42 | 86.55 |
| R12 | 2.49 | 14.43 | 25.58 | 43.99 | 71.41 |
| R13 | 12.90 | 20.82 | 35.92 | 56.12 | 77.04 |
| R14 | 0.00 | 7.95 | 24.13 | 45.02 | 66.20 |
| R15 | 146.27 | 155.04 | 168.66 | 171.21 | 172.24 |
| R16 | 135.64 | 144.15 | 158.08 | 161.26 | 162.38 |
| R17 | 59.84 | 70.55 | 85.96 | 93.57 | 94.73 |
| R18 | 33.55 | 45.05 | 61.55 | 69.99 | 71.17 |
| R19 | 33.55 | 45.05 | 61.45 | 69.99 | 71.17 |
| R20 | 21.59 | 33.51 | 50.58 | 59.22 | 60.39 |
| R21 | 21.59 | 33.52 | 50.51 | 59.22 | 60.39 |
| R22 | 35.73 | 44.45 | 68.78 | 219.05 | 1534.21 |
| R23 | 0.00 | 9.51 | 34.28 | 185.53 | 1500.00 |
| R24 | 10.19 | 10.19 | 10.38 | 12.14 | 12.23 |
| R25 | 0.12 | 0.14 | 0.16 | 0.19 | 0.20 |
| R26 | 0.12 | 0.13 | 0.16 | 0.19 | 0.20 |
| R27 | 0.07 | 0.07 | 0.08 | 0.10 | 0.10 |
| R28 | 0.00 | 0.01 | 0.02 | 0.03 | 0.06 |
| R29 | 0.00 | 0.01 | 0.02 | 0.03 | 0.06 |
| R30 | 160.78 | 200.17 | 270.36 | 409.88 | 747.32 |
| R31 | 0.00 | 3.93 | 37.42 | 160.03 | 486.23 |
| R32 | 7.60 | 7.60 | 7.77 | 9.26 | 9.34 |
| R33 | 2.59 | 2.59 | 2.62 | 2.88 | 2.89 |
| R34 | 0.15 | 0.15 | 0.20 | 0.21 | 0.21 |
| R35 | 0.76 | 0.76 | 0.76 | 1.00 | 1.00 |
| R36 | 1.68 | 1.68 | 1.69 | 1.79 | 1.80 |
| R37 | 9.09 | 9.09 | 9.10 | 10.05 | 10.17 |
| R38 | 0.00 | 0.01 | 0.02 | 0.03 | 0.06 |
| R39 | 0.07 | 0.07 | 0.08 | 0.10 | 0.10 |
| R40 | 1.52 | 1.52 | 1.52 | 1.70 | 1.70 |
| R41 | 2.27 | 2.27 | 2.27 | 2.57 | 2.57 |
| R42 | 6.53 | 6.53 | 6.53 | 7.31 | 7.31 |
| R43 | 1.97 | 1.97 | 2.20 | 2.21 | 2.21 |
| R44 | 0.96 | 0.96 | 1.04 | 1.08 | 1.08 |
| R45 | 9.62 | 9.62 | 10.58 | 10.76 | 10.76 |
| R46 | 24.25 | 24.25 | 25.09 | 27.00 | 27.01 |
| R47 | 12.56 | 12.56 | 12.69 | 13.88 | 13.88 |
| R48 | 23.57 | 23.57 | 23.79 | 26.12 | 26.45 |
| R49 | 2.11 | 2.11 | 2.36 | 2.36 | 2.36 |
| R50 | 1.97 | 1.97 | 2.20 | 2.21 | 2.21 |
| R51 | 1.97 | 1.97 | 2.20 | 2.21 | 2.21 |
| R52 | 0.96 | 0.96 | 1.08 | 1.08 | 1.08 |
| R53 | 0.60 | 0.60 | 0.67 | 0.67 | 0.67 |
| R54 | 0.40 | 0.40 | 0.45 | 0.45 | 0.45 |
| R55 | 0.40 | 0.40 | 0.45 | 0.45 | 0.45 |
| R56 | 0.40 | 0.40 | 0.45 | 0.45 | 0.45 |
| R57 | 0.40 | 0.40 | 0.45 | 0.45 | 0.45 |
| R58 | 0.40 | 0.40 | 0.45 | 0.45 | 0.45 |
| R59 | 1.50 | 1.50 | 1.68 | 1.68 | 1.68 |
| R60 | 3.27 | 3.27 | 3.65 | 3.66 | 3.66 |
| R61 | 3.27 | 3.27 | 3.65 | 3.66 | 3.66 |
| R62 | 6.08 | 6.08 | 6.13 | 6.86 | 6.91 |
| R64 | 6.11 | 7.02 | 8.07 | 8.87 | 10.83 |
| R65 | 10.88 | 11.05 | 11.08 | 12.20 | 12.38 |
| R66 | 19.90 | 20.30 | 20.31 | 22.50 | 22.79 |
| R67 | 9.17 | 9.22 | 10.15 | 10.31 | 10.36 |
| R68 | 7.42 | 7.42 | 8.31 | 8.32 | 8.32 |
